# Repressing CD147 is a novel therapeutic strategy for malignant melanoma

**DOI:** 10.18632/oncotarget.15709

**Published:** 2017-02-25

**Authors:** Xing Hu, Juan Su, Youyou Zhou, Xiaoyun Xie, Cong Peng, Zhimin Yuan, Xiang Chen

**Affiliations:** ^1^ Department of Dermatology, Xiangya Hospital, Central South University, Changsha, China; ^2^ Hunan Key Laboratory of Skin Cancer and Psoriasis, Xiangya Hospital, Central South University, Changsha, China; ^3^ Department of Genetics and Complex Diseases, Harvard T.H Chan School of Public Health, Boston, MA, USA

**Keywords:** cyclophilin A, CD147, melanoma, cell proliferation, MMPs

## Abstract

CD147/basigin, a transmembrane protein, is a member of the immunoglobulin super family. Accumulating evidence has revealed the role of CD147 in the development and progression of various cancers, including malignant melanoma (MM). MM is a malignancy of pigment-producing cells that causes the greatest number of skin cancer-related deaths worldwide. CD147 is overexpressed in MM and plays an important role in cell viability, apoptosis, proliferation, invasion, and metastasis, probably by mediating vascular endothelial growth factor (VEGF) production, glycolysis, and multi-drug resistance (MDR). As a matrix metalloproteinase (MMP) inducer, CD147 could also promote surrounding fibroblasts to secrete abundant MMPs to further stimulate tumor cell invasion. Targeting CD147 has been shown to suppress MM *in vitro* and *in vivo*, highlighting the therapeutic potential of CD147 silencing in MM treatment. In this review article, we discuss CD147 and its biological roles, regulatory mechanisms, and potential application as a molecular target for MM.

## INTRODUCTION

It is estimated that the annual increase in the incidence rate of melanoma has been approximately 3–7% per year worldwide for Caucasians which has the highest incidence [1]; over 90% of U.S. patients with cutaneous Malignant Melanoma (MM) are non-Hispanic whites. However, Cockburn et al. reported that the rate of MM in Hispanic whites is rising [2, 3], and the melanoma occurrence rates have doubled in all socioeconomic status (SES) groups over the last decade [3]. A “good clinical eye” is an “ABCD” rules method-based approach that can be used to reduce melanoma mortality [4, 5]. Tumor invasiveness, distant metastasis, recurrence, and multi-drug resistance (MDR) make MM a leader of fatalities despite a plethora of treatment options, including surgery, chemotherapy, and radiotherapy [6–8]. Therefore, it is necessary to explore effective methods for improving outcomes.

The mechanisms of malignant melanoma are complicated, but include sunlight exposure and heredity [9, 10]. The pathogenesis of UV light exposure is largely unknown, but it directly leads to DNA mutation and accelerates malignant change through overexpressed growth factors like stem cell factor and fibroblast growth factor. MM's resistance to apoptosis reveals that the cancer has a genetic component [11–13]. Recent studies by our group have shown that CD147 plays a pivotal role in the pathogenesis and progression of MM and other diseases [14–23]. Moreover, CD147 has the potential to serve as a novel therapeutic target for MM with multiple drug resistance. In the last decade, we reported that down-regulation and gene knockdown of CD147 could suppress MM`s proliferation, invasiveness, and metastasis. This approach could also stimulate the production of matrix metalloproteases (MMPs), and change the glycolysis level and interaction with calcium-modulating cyclophilin ligand through a dynamic pathway [24–29]. Recently, our team found that CD147 is expressed in the mitochondria and interacts with NDUFS6 to influence complex I viability of MM cell lines. This process may involve the regulation of oxidative phosphorylation (OXPHOS); as a result, CD147 moves to the cytoplasm from the membrane in MM cells [22]. We also reported that CD147 interacted with TRAF6 through ubiquitination to regulate MM`s invasion and metastasis [23]. Therefore, elucidating the underlying mechanisms of CD147-mediated MM metastasis is clinically significant for improving the treatment of MM. In this review, we summarize CD147′s function in MM and the signaling pathways it activates, and discuss its potential role in MM treatment.

**Table 1:** Major clinical trials or treatments for CD147-based therapy in MM

Disease	Description	Year	References
Sepsis-induced acute renal failure	Inhibition of the cyclophilin receptor CD147 attenuates sepsis-induced acute renal failure	2007	Crit Care Med
Oral squamous carcinoma	Inhibition of CD147 and subsequent XIAP depletion may have an anti-tumor effect through enhancing the susceptibility of cancer cells to apoptosis	2009	Cancer Lett.
Acute Myeloid Leukemia	Co-expression of CD147 and vascular endothelial growth factor may indicate a poor prognosis in acute myeloid leukemia and may be a highly sensitive marker for predicting the clinical outcome of patients	2010	Jpn J Clin Oncol
Jurkat T-Lymphoma	Inhibition of CD147 reduces proliferation, activation, adhesion, and migration in human Jurkat T-Lymphoma cells	2008	Cancer Invest.
Breast cancer	CD147 mediates chemoresistance in breast cancer via ABCG2 by affecting its cellular localization and dimerization	2013	Cancer Lett.
Breast cancer	Thrombin-cleaved COOH-terminal osteopontin peptide binds with Cyclophilin C to CD147, and contributes to in vitro migration and invasion in Murine Breast Cancer	2007	Cancer Res.
Malignant melanoma	CD147-targeting siRNA inhibits cell-matrix adhesion of human malignant melanoma cells by phosphorylating focal adhesion kinase	2012	J Dermatol.
Malignant melanoma	CD147 is involved in the uncharacterized [Ca2+]i signaling pathway that may control melanoma invasion, and metastasis	2013	Cancer Lett
Malignant melanoma	Inhibition of CD147 suppresses the proliferation, invasiveness, and VEGF production of human malignant melanoma cells by down-regulating glycolysis	2009	Cancer Lett
Malignant melanoma	Inhibition of CD147 suppresses the proliferation, invasiveness, and metastatic activity of malignant melanoma	2006	Cancer Res
Malignant melanoma	Depletion of CD147 sensitizes human malignant melanoma cells to hydrogen peroxide-induced oxidative stress	2010	J Dermatol Sci.
Malignant melanoma	CD147 is expressed on melanoma cell and induce cell invasion by stimulating MMPs secretion by fibroblasts	2002	Int J Cancer.
Psoriasis	CD147 is highly expressed on peripheral blood neutrophils from patients with psoriasis and induces neutrophil chemotaxis	2010	J Dermatol.
Psoriasis	A miRNA-492 binding-site polymorphism in CD147 confers risk to psoriasis in Central South Chinese population	2011	Hum Genet.

## BIOLOGY OF CD147

### General information

CD147, the symbol for the human Basigin (Bsg) gene, is a transmembrane glycoprotein with two immunoglobulin-like domains and is part of a family that includes embigin and neuroplastin. Several groups found CD147 independently [30–32]: it is located at p13.3 on chromosome 19 [33]. CD147 was identified as a functional molecule involved in many cellular events, such as the inflammatory processes, as a receptor for cyclophilin A [31], and as a potential participant in HIV infection. Moreover, CD147 was found on the surface of tumor cells [34] and may trigger the production or release of MMPs in surrounding mesenchymal and tumor cells, thereby contributing to tumor invasion. The protein portion of CD147 is 28 kDa, but its high glycosylation increases its molecular weight to 43-66 kDa.

CD147 is an important cell-surface protein. There are three Asn glycosylation sites in the extracellular region [31, 34, 35]. The glycan portion differs according to the CD147 source. This glycosylation difference is responsible for various CD147 weights from different sources. CD147 has two Ig domains in the extracellular region. The more C-terminally located Ig-domain has an interesting characteristic; it has homology to both the V domain and the β-chain of the major histocompatibility complex class II, which has the C domain. The V domain and C domain in IgG are only remotely related.

### The biological function of CD147 in cancer

CD147 was found on the surface of tumor cells and promoted the production of MMPs in neighboring mesenchymal cells, leading to enhanced tumor invasion. CD147 is frequently overexpressed in human cancers, and significantly contributes to malignant phenotypes. Up-regulation of CD147 also has been noted in glioma, laryngeal squamous cell, ovarian, renal cell and skin carcinoma [36–39]. Further, CD147 enhances the invasion and survival of cancer cells in various pathways. Among these, the induction of MMPs [28, 34, 40] and vascular endothelial growth factor (VEGF) [40–42] is important. The former enhances tumor invasion and metastasis, while the latter leads to tumor angiogenesis. Cell surface expression of MCTs is required for tumor cell energy metabolism and contributes to their growth and invasion. Activities related to tumor cell survival include suppression of anoikis [43] and increased drug resistance. Furthermore, CD147 promotes hyaluronan synthesis [44], upregulates the Wnt/b-catenin signaling pathway [45], promotes the epithelial-mesenchymal transition [46], and receives a signal of an osteopontin peptide, together with cyclophilin C [47].

### The relationship between cyclophilin A and CD147

Cyclophilin A (CypA) is the host receptor for the immunosuppressive drug cyclosporin A. It is secreted from cells in response to an inflammatory reaction. CypA interacts with its cellular receptor CD147 to exert multiple functions in chemotaxis and cell signaling cascades [48–50]. These correlations of CypA with tumor pathogenesis have been extensively studied [51, 52]. Campa et al. showed that CypA is the most dominantly expressed protein in non-small cell lung carcinoma [53], while Li et al. demonstrated that CypA plays an important role in pancreatic cancer growth through its interaction with CD147 [54]. CypA is a secreted growth factor induced by oxidative stress, and stimulates the ERK1/2 pathway and cell proliferation in vascular smooth muscle cells (VSMCs) [55, 56]. The cytosolic concentration of CypA in T-cell acute lymphocytic leukemia and in mucosal cells from colonic tumors was higher than that in the normal cells. In addition, secreted CypA is chemoattractive to neutrophils, eosinophils, and T cells. Moreover, CD147 has been proposed as a receptor for cyclophilin A [57]. CD147 binds to cyclophilin A and transmits a signal to trigger chemotaxis, and Bsg is involved in signaling of a related protein. The involvement of CD147 in inflammation through binding with cyclophilin A is consistent with previous findings: CD147 becomes expressed in activated lymphocytes, CD147 is upregulated upon collagen-induced arthritis [58], and CD147-deficient lymphocytes exhibit an altered reaction upon mixed lymphocyte reaction.

## THE ROLE OF CD147 IN MALIGNANT MELANOMA

### CD147 regulates cell proliferation and apoptosis of MM cells

It has been well established that CD147 plays critical roles in mediating cell proliferation, apoptosis, and oxidative stress. Miho Hatanaka found melanoma cell growth was inhibited by CD147 silencing [59], Luo Z reported CD147 regulates mitochondrial apoptotic pathway in human malignant melanoma cells by interacting with NDUFS6 [22]. And blocking CD147 induces cell death in cancer cells through impairment of glycolytic energy metabolism [60].Depletion of CD147 sensitizes human malignant melanoma cells to hydrogen peroxide-induced oxidative stress [16]. H2O2 has been shown to induce senescence in normal human skin fibroblasts, in our group we investigated the molecular influences and mechanisms of CD147 on H2O2-induced cellular senescence. We showed that under normal conditions, shRNA-mediated CD147 silencing could inhibit cell proliferation, induce premature senescence, and induce senescence-related cell cycle arrest. While under oxidative stress conditions induced by H2O2, CD147 silencing exacerbated cellular senescence by increasing ROS accumulation and destroying the intrinsic antioxidant defenses. This process might be related to the klotho protein, a newly discovered anti-aging protein [16]. Since CD147 could promote the production and secretion of MMPs from skin fibroblasts, which is the major component of the microenvironment of MM [27], CD147 is potentially implicated in the development and progression of MM.

Accumulating evidence has gradually revealed the role of CD147 in the regulation of biological processes, including cellular viability, apoptosis, senescence, and oxidative stress, of MM cells. For instance, CD147 has been suggested to exert antioxidant activities. We investigated the effects of CD147 on proliferation, apoptosis, and the state of MM cells under H_2_O_2_-induced oxidative stress, and found that inhibition of CD147 increased cellular ROS and destroyed the intrinsic antioxidant defenses in A375 MM cells [26]. Our group showed that CD147 is not only located in cell membrane, but also in the cytoplasm by oxidative phosphorylation (OXPHOS) possibility [22]. This suggests that CD147 has a suppressive effect on H_2_O_2_-induced oxidative damage, and therefore protects against MM cell apoptosis.

### CD147 regulates angiogenesis of MM cells

Angiogenesis plays a crucial role in the invasion and metastasis of malignant tumors [61], and CD147 has been reported to induce angiogenesis in pathological processes, including cancers [62–64]. Hatanaka M found CD147 regulates the angiogenesis by decreasing VEGF expression *in vivo* and reducing blood vessel formation [65]. Our group has demonstrated that the co-expression of CD147 and VEGF might indicate a poor prognosis in acute myeloid leukemia and may be a highly sensitive predictor of clinical outcome [66]. Furthermore, CD147 was found to participate in the regulation of angiogenesis in MM. Su et al. showed that CD147 silencing could not only suppress MM cell proliferation and invasion, but also inhibited the production of VEGF in MM cells *via* downregulation of monocarboxylate transporters (MCT) 1 and MCT4. These transporters mediate lactate transport, suggesting that CD147 may promote tumor cell glycolysis and progression of MM through interacting with MCT1 and MCT4 [25]. We also demonstrated that siRNA-mediated CD147 silencing inhibited the expression of VEGF in MM cells and decreased endothelial cell migration, which is closely related to the invasion and metastasis of MM [29]. More importantly, we established a nude mouse xenograft model of MM and showed that downregulation of CD147 could suppress the tumor's size and microvessel density [29].

### CD147 regulates cancer invasion and metastasis in MM

Voigt H found CD147 impacts metastasis formation [65]. And blocking CD147 could inhibit the invasiveness, and metastatic activity of malignant melanoma [29]. In our group we recently showed that the endoplasmic reticulum (ER) -associated protein calcium-modulating cyclophilin ligand (CAML) is bound to CD147 in human A375 melanoma cells. CD147 silencing significantly decreased resting [Ca2+]i and the [Ca2+]i increase induced by the sarco/endoplasmic reticulum Ca2+-ATPase (SERCA) inhibitor thapsigargin (TG), indicating that the interaction between CAML and CD147 regulates ER-dependent [Ca2+]i signaling. Upregulation of [Ca2+]i could induce the production of MMP-9 in A375 cells with the expression of CD147 [24]. Thus, CD147 may participate in the ER-dependent [Ca2+]i signaling pathway, which may mediate MM invasion and metastasis.

Thus, CD147 may act as an oncogene in MM, and targeting CD147 could inhibit cancer cell viability, proliferation, and invasion, while inducing cell senescence and apoptosis in MM cells. The underlying molecular mechanism responsible for this may be CD147′s regulation of oxidative stress, glycolysis, and angiogenesis in either the MM cells or surrounding cells in the tumor microenvironment.

**Figure 1:**
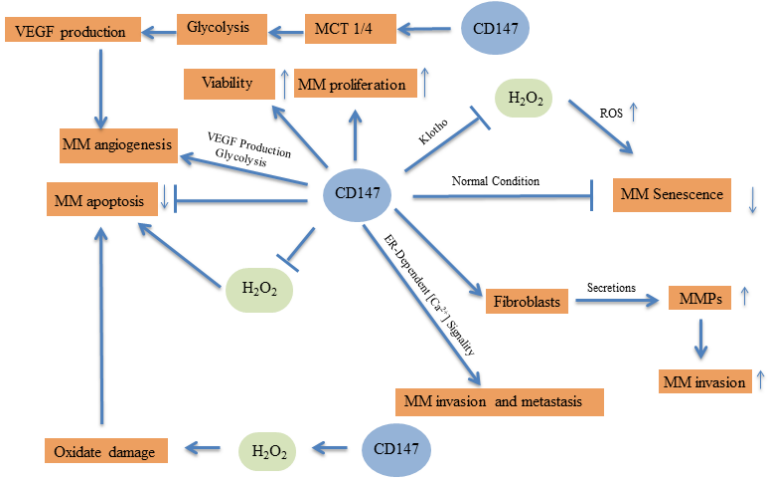
Molecular pathways of CD147 in malignant melanoma

## THERAPEUTIC POTENTIAL

Since CD147 plays a role in various diseases, it has recently been suggested as a promising target for the treatment of various diseases. For instance, liver sepsis could induce a universally altered profile of liver proteins, including increased cyclophilin. However, inhibition of CD147, the receptor of cyclophilin, could effectively attenuate sepsis-induced acute renal failure *via* inducing a significant reduction in serum cytokine production [67]. Thus, CD47-targeted therapy may help prevent sepsis-induced renal dysfunction. Furthermore, CD147 has been suggested to play a role in psoriasis. We showed that CD147 is highly expressed on peripheral blood smears and induces neutrophil chemotaxis [18]. We also found a miRNA-492 binding-site polymorphism in CD147. This conferred psoriasis risk upon the central south Chinese population, and suggested that this polymorphism might be associated with reduced psoriasis susceptibility, by affecting miRNA-492 binding [17]. Additionally, we suggested a role of CD147 in regulating ABCG2 transport of methotrexate in immune cells [15]. Therefore, strategies involving CD147 targeting could be considered for the clinical treatment of patients with psoriasis that is resistant to methotrexate. These findings emphasize the significance of CD147 in the development of psoriasis.

CD47 silencing also has applications in the treatment of malignant tumors. We found that CD147 had implications in the regulation of drug transport by mediating the expression and dimerization of ABCG2. This affected ABCG2′s cellular localization and drug transporter function in breast cancer cells [14]. In addition, we found that siRNA-mediated CD147 inhibition could reduce proliferation, activation, adhesion, and migration in human Jurkat T-lymphoma cells [21]. We also investigated the effect and mechanisms of CD147 on the MDR phenotype of human oral squamous carcinoma cells (SCCs), and showed that the expression of CD147 and X-linked inhibitor of apoptosis (XIAP) was upregulated in MDR-derivative SCCs compared with SCCs. We further revealed that inhibition of CD147 and subsequent XIAP depletion might have an anti-tumor effect through enhancing the susceptibility of cancer cells to 5-fluorouracil-induced apoptosis [20]. Since SCC and MM are both cutaneous carcinomas, targeting CD147 may also become a potential therapeutic strategy for the treatment of MM. Further, Chen and colleges reported that targeting CD147 could effectively suppress the size and microvessel density of tumors in a nude mouse xenograft model of MM. In addition, the *in vivo* metastatic potential of A375 cells transfected with CD147 siRNA was suppressed in a nude mouse model of pulmonary metastasis [29]. Accordingly, CD147 may become a potential therapeutic target for the treatment of MM, mainly because of its regulatory role in chemical resistance and tumor metastasis.

## CONCLUSIONS

Despite recent progress on the diagnostic and therapeutic aspects of MM, the overall survival rate of patients with MM remains unchanged. Emerging evidence has gradually revealed the genomic information and interaction of signaling networks in MM cells, shedding new light on how to tailor an effective treatment. New emerging targets, such as CD147, can also be used to assess the prognosis and risk of recurrence in MM. However, further studies are needed to unravel the detailed mechanism of the CD147 pathway in MM and other cancers.
